# Repopulated retinal microglia promote Müller glia reprogramming and preserve visual function in retinal degenerative mice

**DOI:** 10.7150/thno.79538

**Published:** 2023-03-13

**Authors:** Xuan Cheng, Hui Gao, Zui Tao, Zhiyuan Yin, Zhe Cha, Xiaona Huang, Yikui Zhang, Yuxiao Zeng, Juncai He, Lingling Ge, Luodan A, Haiwei Xu, Guang-Hua Peng

**Affiliations:** 1 Department of Ophthalmology, First medical center of Chinese PLA General Hospital, Beijing, 100853, China.; 2 Southwest Hospital/Southwest Eye Hospital, Third Military Medical University (Army Medical University), Chongqing 400038, China.; 3 Key Lab of Visual Damage and Regeneration & Restoration of Chongqing, Chongqing 400038, China.; 4 The Eye Hospital, School of Ophthalmology & Optometry, Wenzhou Medical University, Wenzhou 325027, China.; 5 Lab of Visual Cell Differentiation and Regulation, Basic Medical College, Zhengzhou University, Zhengzhou, China.

**Keywords:** Microglia, Müller glia, reprogramming, repopulation, extracellular matrix

## Abstract

**Rationale:** Müller glia (MG) play a key role in maintaining homeostasis of the retinal microenvironment. In zebrafish, MG reprogram into retinal progenitors and repair the injured retina, while this MG regenerative capability is suppressed in mammals. It has been revealed that microglia in zebrafish contribute to MG reprogramming, whereas those in mammals are over-activated during retinal injury or degeneration, causing chronic inflammation, acceleration of photoreceptor apoptosis, and gliosis of MG. Therefore, how to modulate the phenotype of microglia to enhance MG reprogramming rather than gliosis is critical.

**Methods:** PLX3397, a colony-stimulating factor 1 receptor inhibitor, was applied to deplete microglia in the retinas of retinal degeneration 10 (rd10) mice, and withdrawal of PLX3397 was used to induce the repopulated microglia (Rep-MiG). The protective roles of the Rep-MiG on the degenerative retina were assessed using a light/dark transition test, and scotopic electroretinogram recordings. Immunofluorescence, western blot, transcriptomic sequencing, and bioinformatics analysis were performed to investigate the effects and mechanisms of microglia on MG reprogramming.

**Results:** Following PLX3397 withdrawal, Rep-MiG replenished the entire retina with a ramified morphology and significantly improved the retinal outer nuclear layer structure, the electroretinography response, and the visual behavior of rd10 mice. Coincidentally, MG were activated, de-differentiated, and showed properties of retina progenitors in a spatial correlation with Rep-MiG. Morphological and transcriptomic analyses revealed Rep-MiG significantly enhanced protease inhibitor activity and suppressed extracellular matrix (ECM) levels during retinal degeneration.

**Conclusions:** It suggested that Rep-MiG with the homeostasis characteristic stimulated the progenitor cell-like properties of MG, probably through regulating ECM remodeling, which protected photoreceptors and improved visual function of rd10 mice. It might be a potential protocol to reprogram MG and delay mammal retinal degeneration.

## Introduction

Among retinal damaging diseases, retinitis pigmentosa (RP) is usually inherited and affects more than 1/4000 people worldwide 1. RP is mainly characterized by the death of primary rod and secondary cone photoreceptors, leading to irreversible visual loss. There are currently no effective treatments for RP. Preserving residual photoreceptors or regenerating new photoreceptors are considered to be key in rescuing visual function 2, 3. Different from the multiple risks of transplanting exogenous stem cells, activation of endogenous stem cells has been recently regarded as a more promising strategy to supplement lost neurons in the central nervous system (CNS).

In the brain, radial glial cells respond to various injuries by proliferating and generating neurogenic progenitors and regenerating neurons 4. As special radial glial cells, Müller glia (MG) play critical roles in maintaining the retinal structural integrity and physiological functions. MG are widely accepted as a candidate for endogenous stem cells for retinal repair 5-7. In zebrafish, as a response to retinal injury, MG de-differentiate into retinal progenitors, proliferate and differentiate into all types of retinal cells 5. However, the regenerative capacity of MG is suppressed in mammals 6. Recently, targeting crucial genes in MG has been shown to achieve MG-mediated retinal regeneration in mammals, although the specificity and efficiency of this technique require further improvement 8-11.

In addition to intrinsic blockades, the extrinsic microenvironment, especially the immune-microenvironment, has been reported to be a critical regulator for MG reprogramming 5. Importantly, activated microglia are considered a central player in regulating the immune-microenvironment 5. In zebrafish, as a reaction to retinal injury, activated microglia are transiently recruited into the injured site, where they engulf cell debris and contribute to MG reprogramming 12. Once MG reprogramming is complete, microglia immediately evacuate the injury sites, rapidly recapitulating the original homeostasis of the normal retina 13. Microglial activation is essential for promoting MG reprogramming in zebrafish by regulating the immune-microenvironment after retinal injury 12, 13. However, in mammals, MG show rapid changes in gene expression at the early stage of retina degeneration before a gradual reversion toward a resting state, ultimately gliosis 11. In our previous studies, microglia are gradually activated into the intermediate stage before being converted to disease-associated microglia, suggesting that there are different subtypes of microglia at the early and late stages of RP 14. Accordingly, diverse subtypes of microglia seem to play different roles in determining the fate of endogenous stem cells during different phases of injury/degeneration 15. Pro-inflammatory microglia aggravate gliosis of neural stem cells, whereas anti-inflammatory microglia promote neurogenesis 16. Another recent study showed that microglia ablation enhances the neurogenic capacity of MG during a combination of ANT treatment-mediated retinal regeneration 17. It is vital to regulate microglia phenotypes according to different inflammatory stages during injury/degeneration to regenerate the injured retina 16-19. Still, it has not yet been clarified whether different microglia phenotypes affect MG reprogramming by regulating the immune-microenvironment.

Recently, as the survival of microglia depends on colony-stimulating factor 1 receptor (CSF1R) signaling, CSF1R inhibitors have been used to eliminate microglia rapidly to elucidate their role in the CNS. Interestingly, microglia can be replenished throughout the retina and brain quickly after the withdrawal of CSF1R inhibitors 20, 21. These repopulated microglia (Rep-MiG) regain the typical ramified morphology of resting microglia, with regular distribution patterns and gene expression profiles, as shown in the healthy retina and brain 22. Rep-MiG downregulate the expression of inflammation-associated genes, thus blocking neuroinflammation, alleviating neurodegeneration, and restoring neural synaptic connectivity, leading to functional recovery in the neurotraumatic and neurodegenerative mouse brain 22, 23. However, it is unclear whether Rep-MiG produce similar neuroprotective and pro-neuroregeneration effects in the degenerative retina.

Here, we demonstrated that Rep-MiG replenished the entire retina following the depletion of microglia using the CSF1R inhibitor PLX3397 in retinal degeneration 10 (rd10) mice, a classic RP model. The Rep-MiG, which had a ramified morphology and were mainly in the inner plexiform layer (IPL), significantly improved the retinal outer nuclear layer (ONL) structure, electroretinography (ERG) response, and visual behavior of rd10 mice. Moreover, Rep-MiG activated the stem cell-like characteristics of MG in a spatially correlated pattern. RNA-Seq and immunofluorescence staining revealed that Rep-MiG with the homeostasis characteristic regulated ECM remodeling during retinal degeneration, which might create a permissive microenvironment to facilitate MG reprogramming. Thus, our results provide a potential protocol to promote MG reprogramming and protect the retina during retinal degeneration by modulating the microenvironment in mammals.

## Materials and Methods

### Animals

c57BL/6 mice (c57 mice) and rd10 mice were provided by the Experimental Animal Center of Army Medical University. Glast-Cre^ER^ transgenic mice (Jackson Laboratory, stock No. 012586) were crossed with Cre-inducible CAG-LSL-tdTomato reporter mice [B6/JNju-H11^em1Cin(CAG-L^°^xP-ZsGreen-St^°^p-L^°^xP-tdT^°^mat^°^)^/Nju, purchased from JiCuiYaoKang Biology Company (Nanjing, China)]. The resulting Glast-Cre^ER^ -loxp positive mice were crossed with rd10 mice to generate Glast-Cre^ER^ -loxp positive rd10 mice (cre-loxp+ rd10 mice). The retinas of cre-loxp^+^ rd10 mice not only degenerate with age according to the original disease pattern of rd10 mice but also induce red fluorescence on MG following systemic injection of tamoxifen. All of the above-mentioned animals were housed in a specific pathogen-free room at the animal center of Southwest Hospital. Mice were fed a standard diet, with water, under a 12-h light/dark cycle. All experimental procedures were approved by the Ethics Committee of Southwest Hospital, Army Medical University, in compliance with the statement for the use of animals from the Association for Research in Vision and Ophthalmology. Only male mice were randomly assigned to experiments, and at least three mice were used for each experiment.

### Tamoxifen injection

Tamoxifen (Sigma, T5648) was dissolved in corn oil and injected intraperitoneally (120 mg/kg body weight) into the cre-loxp^+^ rd10 mice for 4 consecutive days, coupled with another 3 days without treatment to induce the expression of tdTomato.

### Drug administration

**Figure 1:**
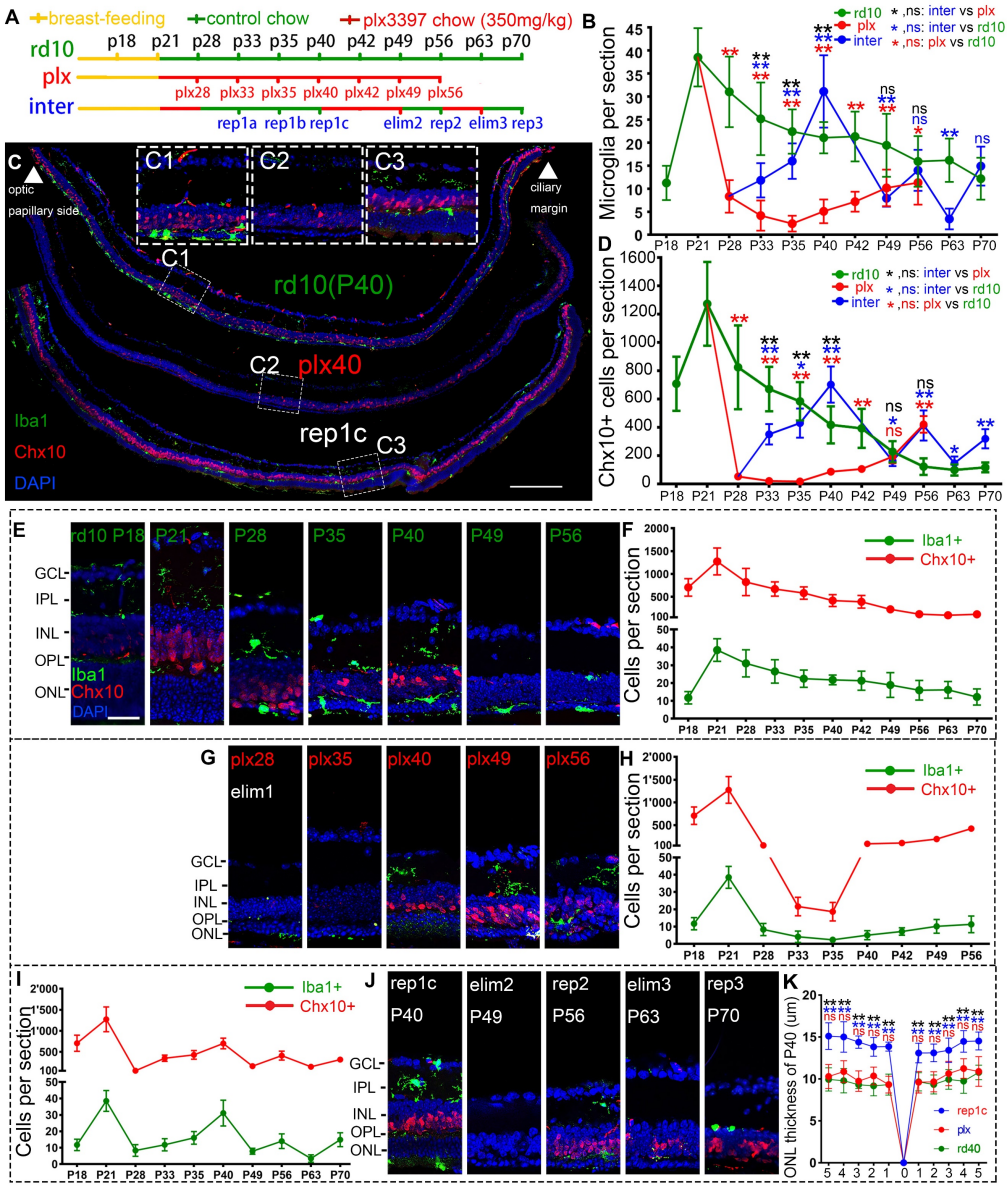
** Rep-MiG increased the number of retinal progenitors and protected photoreceptors of rd10 mice.** (A) Treatments and nomination of each sampling in the rd10, plx, and inter groups. Breastfeeding (yellow), regular feeding (green), and plx3397 feeding (red) were indicated. (B) Enumeration of Iba1^+^ cells in each retinal section from the rd10, plx, and inter groups. (N = 4 mice, n = 2-3 retinal sections per mouse). (C) Distribution of Iba1^+^ and Chx10^+^ cells in rd10(P40), plx40, and rep1c mice; dashed box C1/2/3 is the correspondingly enlarged view in panel C. (D) Counting of Chx10^+^ cells in each retinal section for the rd10, plx, and inter groups. (N = 3 mice, n = 2-3 retinal sections per mouse). (E-J) Schematic representation of changes in Iba1^+^ and Chx10^+^ cells in each retinal section for the rd10 (E, F), plx (G, H), and inter groups (I, J). (K) Statistical analysis of the ONL thickness in the rd10, plx, and inter groups at P40. (N = 3 mice, n = 2 retinal sections per mouse). Data are presented as the mean ± SD. *P < 0.05; **P < 0.01; ns, No significance (one-way ANOVA for B, D, K). Scale bars: 200 μm (c), 20 μm (C1/2/3, E). GCL: Ganglion cell layer, IPL: Inner plexiform layer, INL: Inner nuclear layer, OPL: Outer plexiform layer, ONL: Outer nuclear layer.

According to a previous report 21, rd10 mice were fed a plx3397-formulated AIN-76A diet (350 p.p.m; containing 350 mg of drug per l kg of feed [Selleckchem, USA]) *ad libitum* to eliminate microglia. The rd10 group was the natural course group of rd10 mice. In the plx group, rd10 mice were treated with sustained clearance of microglia via plx3397 feeding; in the inter group (intermediate dosing group), rd10 mice were fed a plx3397-formulated AIN-76A diet from postnatal 21 days (P21) to P28 (marked as elim1), followed by 5 days (P33, rep1a), 7 days (P35, rep1b), 12 days (P40, rep1c), and 20 days (P48, rep1d) of a normal AIN-76A diet for microglial repopulation; rep1c mice were fed a plx3397-formulated AIN-76A diet again at P40 for 9 days to clear microglia for the second round (P49, elim2); elim2 mice were followed by a normal AIN-76A diet for 7 days so that microglia could repopulate for the second time (P56, rep2); and rep2 mice were fed a plx3397-formulated AIN-76A diet for another 7 days to achieve the third round of microglia clearance (P63, elim3), followed by a normal AIN-76A diet for 7 days for the third round of microglia repopulation (P70, rep3) (Figure 1A).

### Immunohistochemistry

For immunofluorescence staining 24, mice were euthanized and their eyes were removed. The eye cups were separated and enucleated, fixed with 4% paraformaldehyde (PFA) at 4 °C for 30 min, followed by overnight dehydration in 30% glucose solution. After embedding in tissue embedding agent optimal cutting temperature (OCT) compound, the eyecups were cut into 12 μm-thick sections via a cold microtome and were attached to glass slides. The intact retina sections containing or adjacent to the optic disc were preferentially selected for immunohistochemistry staining. PT1 (PBS containing 0.1% Triton X-100 and 10% goat serum) was used to permeabilize and block the sections at 37 °C for 1 h. The sections were incubated with primary antibodies in PBS containing 0.03% Triton X-100 and 5% goat serum overnight at 4 °C. The primary antibodies and dilutions are shown in Table 1. The next day, after three washes with PBS, the sections were incubated with secondary antibodies (Table 1) for 1 h at 37 °C, and the nuclei were counterstained with DAPI (Sigma-Aldrich). Images of immunofluorescence staining were viewed and photographed via a confocal laser scanning microscope (Zeiss LSM 780). Positive cells were counted manually using a panoramic view of retinal sections, preferably through or near the myopic papilla.

### Outer nuclei layer (ONL) thickness analysis

Each panoramic retinal view was obtained from the tiling of 6-7 individual graphs stained with DAPI at 200× magnification. For each group, 3 mice at each time point and 2 sections passing through or near the optic papilla were used for immunostaining analysis. We defined the optic disc as the original location (recorded as 0) and selected ten positions uniformly at both the temporal and nasal sides of the retina. The ONL thickness was analyzed based on its vertical length measured using ImageJ as previously described 25.

### Grid analysis of microglia

Analysis of retinal microglia was performed as previously reported 24. In brief, nine retinal sections from three mice per group were stained with Iba1. Using a Zeiss confocal imaging system, the number of grid cross points of each microglia was analyzed. The relative crossing frequency in each group at d levels was fulfilled based on the averaged FPKM values of genes in the retina samples from c57 (P40), rd10 (P40), plx40, and rep1c mice. Differently expressed genes (DEGs) were those with a fold change in expression > 1.2 and *P* value < 0.05. Gene Ontology (GO) and Kyoto Encyclopedia of Genes and Genomes (KEGG) enrichment of DEGs were analyzed using the databases. Gene Set Enrichment Analysis (GSEA) was conducted by GSEA 4.2.3 and items with |NES| > 1, *P* < 0.05, and false discovery rate (FDR) < 0.25 were statistical significance.

### Western blot

Retina samples were isolated from mice of different groups at various time points. Fresh frozen retina solution was diluted in 50 µL PBS in a cold environment. Tissue lysis buffer (10% PMSF and 90% RIPA) was added to extract protein, and the concentration was measured using a BCA assay. The protein was loaded and separated on a 12% SDS-PAGE gel before being transferred to polyvinylidene fluoride (PVDF) membranes. Electrophoresis was performed at 80 V for stacking and 100 V for separation. After blocking with 5% skim milk in TBST buffer (20 mM Tris base, 150 mM NaCl, and 0.1% Tween 20) for 1 h at 37 °C, membranes were incubated with primary antibodies, consisting of rabbit anti-GFAP (Abcam, ab48050, 1:2000), and mouse β-actin (Immunoway YT0099, 1:2000), overnight at 4 °C. The next day, the membranes were incubated with HRP-conjugated secondary antibodies for 1 h at room temperature. Probed using Pierce ™ ECL Western blotting substrate (Thermo, 32106), proteins on the membrane were scanned using an exposure system (bioRAD). Relative expression levels were determined using ImageJ software (NIH) with β-actin as a control.

### Scotopic electroretinogram (ERG) recordings

According to a previous protocol 24, mice were dark-adapted for 12 h before being anesthetized under dim red light (wavelength > 620 nm). The pupils of the mice were dilated using 1% tropicamide for ERG recording, and light stimuli were at intensities of 0.01 cd·s /m^2^, 0.1 cd·s /m^2^, 1.0 cd·s /m^2^, 3.0 cd·s /m^2^, 5.0 cd·s /m^2^ and 10.0 cd·s /m^2^. To avoid hypothermia, the body temperature of mice was maintained at 37 °C using a heated mat. Two active gold electrodes were attached to each cornea to record the corneal ERG responses. The reference and ground electrodes were embedded subcutaneously in the mid-frontal regions of the head and tail, respectively. The interstimulus interval varied from 30 to 120 s based on light intensity. The response of each flash, and the amplitudes of a- and b-waves, were recorded using an RETI-Port device (MAYO), in which the a-wave amplitude was gauged based on the maximum negative trough below the baseline, while b-wave amplitude as the difference from the a-wave trough to the subsequent positive peak. The acquired data were processed using Igor software.

### Light/dark transition test

Visual behavior tests were performed using a previously described protocol 26. Briefly, the test box contained a dark and light chamber linked by an open door. The test mouse was placed in the dark chamber and freely moved in the shuttle box. The movement trajectories were recorded using a video camera mounted in the chamber. The time and ratio of mice spent in the dark or light chamber during the 10-min test were determined via manual timing using the time-meter function of Adobe Premiere Pro.

### Statistics

Data are presented as the mean ± standard deviation (SD) of at least three independent biological samples. Data were analyzed through one-way analysis of variance (ANOVA) using Statistical Product and Service Solutions software V25.0 (SPSS V25.0). Differences were considered statistically significant at *P* < 0.05.

## Results

### Rep-MiG increased the number of retinal progenitor-like cells that protected photoreceptors in rd10 mice

Rd10 mice received the AIN-76A diet ad libitum to eliminate microglia, then plx3397 was withdrawn to induce Rep-MiG in the intermediate dosing group (inter group). Different dosages of plx3397 (290 mg/kg, 350 mg/kg, and 600 mg/kg) were administered to rd10 mice to determine the most appropriate concentration for microglial clearance. After one week of treatment with plx3397 at the three dosages, the microglia number in the rd10 mouse retinas at P28 was reduced by 64.2%, 78.3%, and 74.3%, respectively (data not shown). Therefore, we utilized plx3397 at 350 mg/kg to clear the microglia of rd10 mice at P21 when the mice were weaned and one week after the beginning of retinal degeneration. The administration pattern and each sampling nomination in the three groups (rd10, plx, and inter) are shown in Figure 1A. Iba1 is a cell marker of microglia and Chx10 denotes retinal progenitors. In the rd10 group, the Iba1^+^ microglia number increased markedly at the initiation of degeneration (P15) and peaked on P21. Subsequently, the Iba1^+^ microglia number gradually decreased but remained at more than 15 per section from P28 to P63, and more than half of Iba1^+^ microglia were in the ONL and subretinal space (SRS) (Figure 1B, E, F). In the plx group, one week of the plx3397 diet from P21 reduced the microglia number by 78.3% at plx28, while two weeks reduced the number by 93.7% at plx35 before starting to resurge (Figure 1B, G, H). The resurgent microglia reappeared around P40 and increased persistently until P56 (Figure 1G, H), in line with the trend of microglia counts in our previous study on Royal College of Surgeons (RCS) rats 27. As the microglia reappeared and proliferated against the use of a microglia depletion drug, they were termed drug-resistant microglia (Dr-MiG). In the inter group, microglia started to recolonize after plx3397 withdrawal on P28 (the first round of repopulation) with a progressively increased number from P33 to P40 (Figure 1B, J). Following nine days of plx3397 administration to rep1c mice, the number of microglia dropped to 7.9 in elim2 (second round of elimination) mice. After plx3997 withdrawal for seven days, the microglia number increased slightly to 14.0 in rep2 (second round of repopulation) mice. Then, after plx3397 administration for seven days, the number of microglia in elim3 (third round of elimination) mice reduced to 3.5. Finally, in the third round of microglia repopulation, the number increased to 14.9 in rep3 (third round of repopulation) mice (Figure 1B, I, J).

More interestingly, the number of Chx10^+^ cells swayed as degeneration progressed, particularly as the number of Iba1^+^ microglia fluctuated (Figure 1B, D). In the rd10 group, Chx10^+^ cells rose from P18 to P21 and then gradually declined until P56 during the progression of retinal degeneration (Figure 1D, F). At P56 in rd10 mice, Chx10^+^ cells were mainly in the ciliary marginal zone (CMZ) where retinal progenitors were located 28. (Figure 1E, F, Figure S2). In the plx group, Chx10^+^ cells rapidly decreased at the time of microglia removal (plx28) and continued until plx35 (Figure 1D) before gradually increasing with the recurrence of Dr-MiG (Figure 1G, H). In the inter group, the alterations in the number of Chx10^+^ cells were consistent with the swings in the number of microglia (Figure 1I, J). The distributions of Iba1^+^ and Chx10^+^ cells in the peripheral, middle peripheral, and middle retinas at different time points were shown in Figure S1. Strikingly, the numbers of Iba1^+^ and Chx10^+^ cells in the inter group peaked and were significantly increased compared to the rd10 and plx groups at P40 except for the initial degeneration (Figure 1B, D).

Thus, to compare the influences of different subtypes of microglia on the retinal progenitors and visual function of rd10 mice, P40 was selected as the unique time point to analyze the rd10, plx, and inter group, namely, rd10 (P40), plx40, and rep1c mice (Figure 1C). As degeneration progressed, the Chx10^+^ cell density outside the CMZ declined markedly from P21 to P40 (Figure 1E, F). Correspondingly, considerably more branched iba1^+^ microglia were in the inner retina of the rd10 (P21) mice compared to the rd10 (P40) mice, whose microglia were more amoeba-like seated in the outer retina (Figure 1E). In the plx group, i.e., in plx40 mice, the Chx10^+^ cells were distributed in the CMZ, as well as scattered near Iba1^+^ microglia, and the number was significantly decreased compared to that of contemporaneous rd10 (P40) mice (Figure 1G, H, Figure S1B). However, in the inter group, only a few Chx10^+^ cells were located in the CMZ in the retinas of plx28 mice (Figure S1C). Yet, 12 days post plx3397 withdrawal, in rep1c mice, microglia were repopulated panretinally, with 71% of the Rep-MiG located in the IPL and outer plexiform layer (OPL). Massive panretinal recurring of Chx10^+^ cells in the retina was in rep1c mice (Figure 1I, J, Figure S1F). At P40, the difference in ONL thickness among rd10 (P40), rep1c, and plx40 mice was statistically significant (Figure 1K). Meanwhile, the thickness of the inner nuclei layer (INL) in rep1c mice was significantly thicker than that in rd10 (P40) and plx40 mice (data not shown). These results imply that Rep-MiG activate retinal progenitors in rd10 mice, and exert a neuroprotective effect on photoreceptors.

### Spatial correlation between Rep-MiG and retinal progenitors in rd10 mice

The Chx10^+^ cells in non-CMZ regions changed with the amount and distribution of microglia, which did not appear in the CMZ. There was no significant difference in the Chx10^+^ cell count of the CMZ among the three groups at various time points (Figure S2).

**Figure 2:**
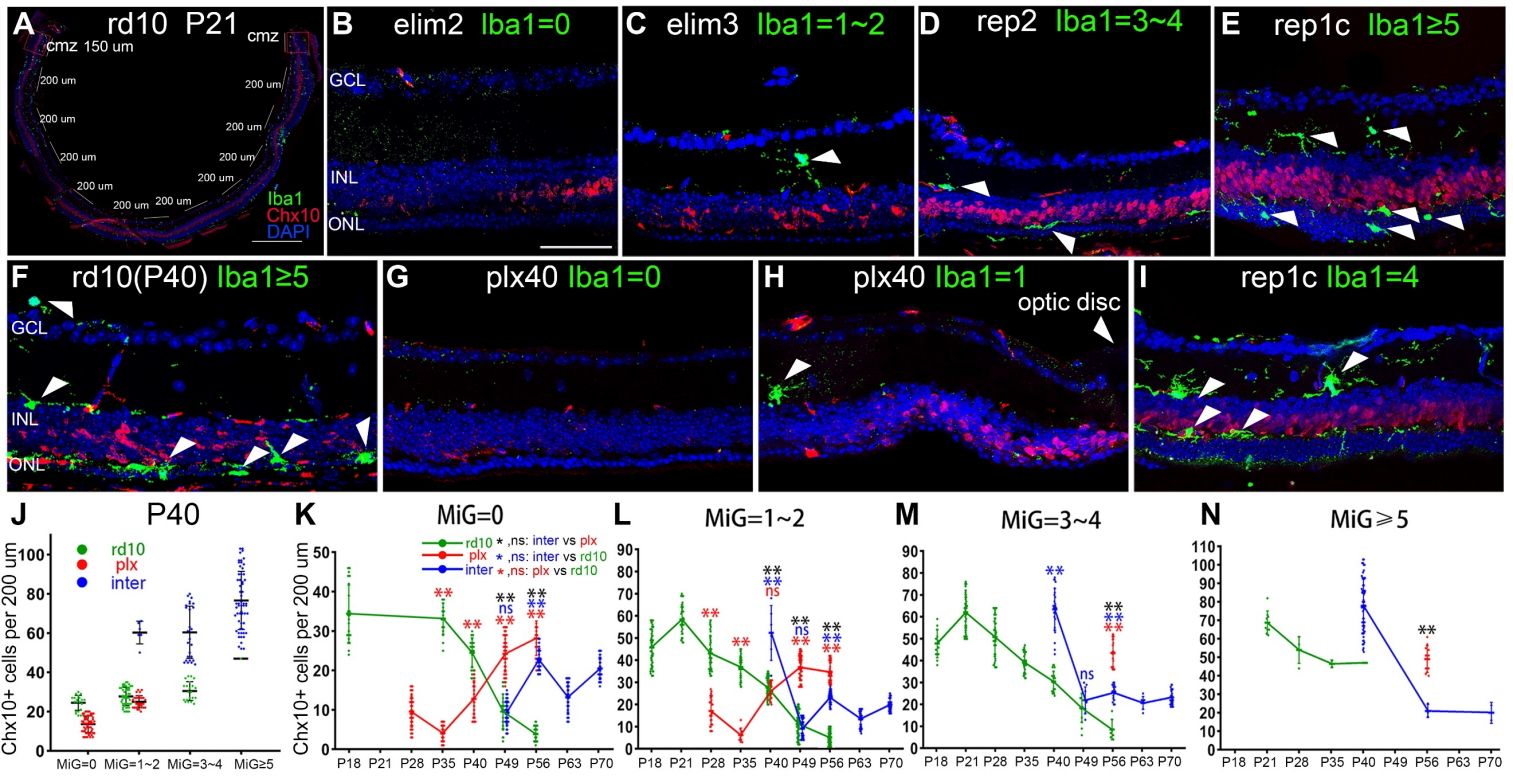
** Spatial correlation between retinal microglia and retinal progenitor-like cells in the retinas of rd10 mice.** (A) In each tiled up panoramic retinal section, except for the 150-long CMZs and the papillary region, 10 retinal segments with a length of 200-μm were randomly specified. The number of Iba1^+^ cells and Chx10^+^ cells were counted in each retinal segment. (B-E) The typical retinal segments from the inter group with 0, 1-2, 3-4, or ≥ 5 Iba1^+^ cells (green). White arrowheads indicate individual microglia. (F-I) Typical retinal segments from the three groups at P40, with panels G and H showing the mid-peripheral area and the area close to the myopic papilla retina, respectively. (J) Counts of Chx10^+^ cells in the mice retina segments of three groups at P40, with 0, 1-2, 3-4, or ≥ 5 Iba1^+^ cells) (N = 3 mice, n =3 retinal sections per mouse). (K-N) Counts of Chx10^+^ cells in the mice retina segments of the three groups at different time points, with 0, 1-2, 3-4, or ≥ 5 Iba1+ cells. (N = 3 mice, n = 3 retinal sections per mouse). Data are presented as the mean ± SD, *P < 0.05; **P < 0.01; ns, No significance (one-way ANOVA for J-N). Scale bars: 400 μm (A), 40 μm (B-I). GCL: Ganglion cell layer, INL: Inner nuclear layer, ONL: Outer nuclear layer.

Moreover, clearing or recolonizing microglia did not substantially affect the CMZ stem cell pool throughout the disease in rd10 mice. Therefore, the following studies excluded CMZ. To further analyze the spatial correlation between Rep-MiG and retinal progenitors, each retina, apart from the two 150-μm long CMZs, was divided into ten segments (200 μm long), in which we counted Iba1^+^ microglia and Chx10^+^ retinal progenitors (Figure 2A). Specifically, the segments were classified into four categories according to microglia counts. At P40, the number of Chx10^+^ cells sequentially increased concomitantly with increased microglia number. The markedly increased Chx10^+^ cells were in the inter group than the rd10 and plx groups in regions with the same microglia number at P40 (Figure 2F-J).

However, the findings varied slightly among the rd10, plx, and inter groups (Figure 2K-N). In the rd10 group, the Chx10^+^ cell count began to decline after P21, regardless of the rising segment of amoeba-like microglia. Most Iba1^+^ microglia were ameboid and distributed in the outer retina after P28, and these hyperactivated microglia possibly did not support the progenitor-like phenotype. In the plx group, at time points after P35, the Chx10^+^ cell counts also displayed an upward tendency, accompanied by the appearance of Dr-MiG. In the inter group, the number of Chx10^+^ cells repeatedly swayed with the clearing and repopulation of microglia (Figure 2B-E). These results indicate a spatial correlation between diverse microglial subtypes and Chx10^+^ cells and that Rep-MiG induce the recurrence of retinal progenitors in a spatially correlated manner in rd10 mice.

### Rep-MiG improved the visual function of rd10 mice at P40

**Figure 3:**
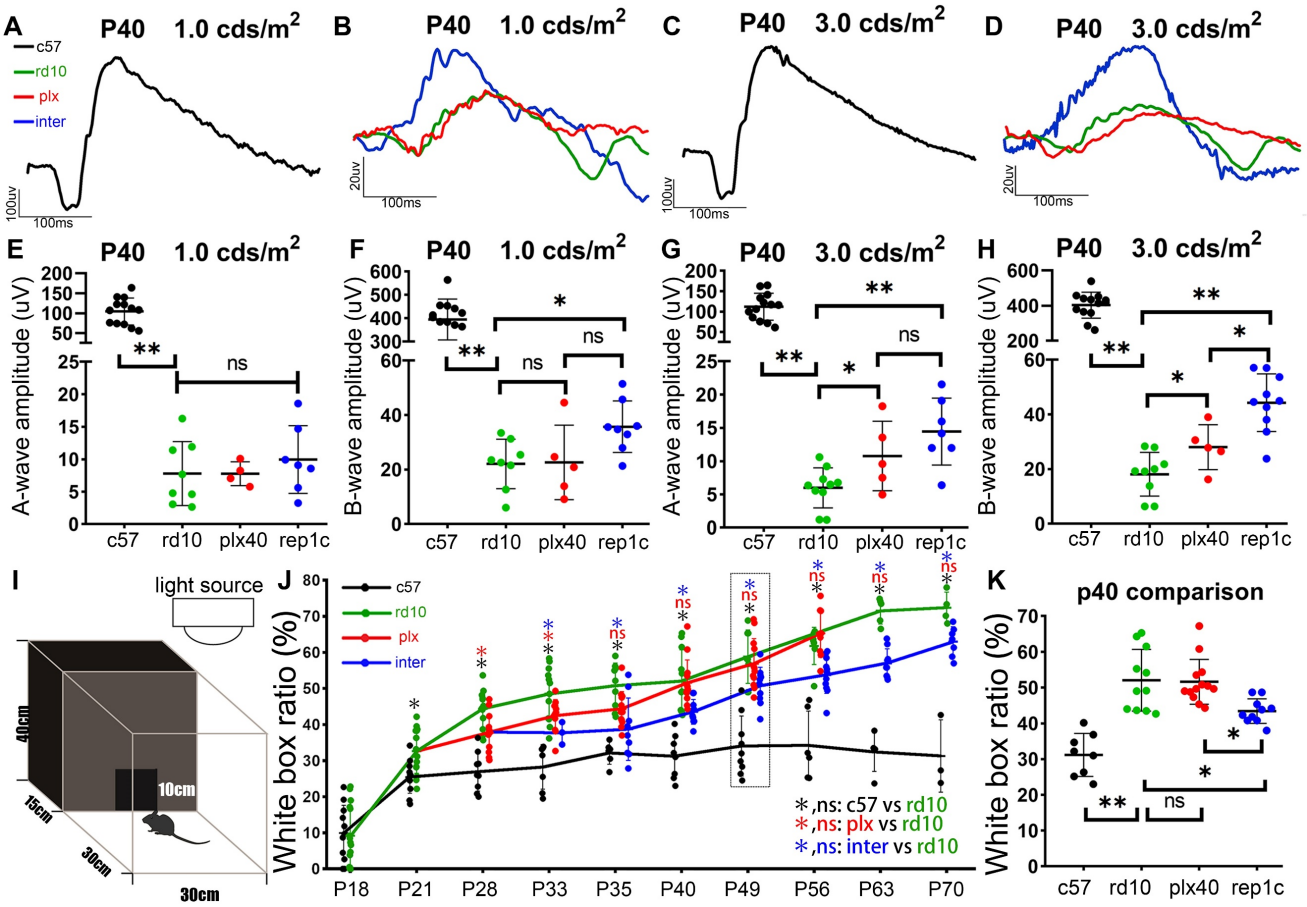
** Rep-MiG improved the visual function and behavioral measures of rd10 mice.** (A-D) Representative flash electroretinograms (fERG) at P40 in c57 (black) (A, C), rd10 (green), plx (red), and inter group (blue) (B, D). (E-H) Amplitudes of the fERG a-wave (E, G) and b-wave (F, H) in c57 (black), rd10 (green), plx (red), and inter (blue) groups at P40, with stimulating light of 1.0 cd·s /cm^2^ (E, F) and 3.0 cd·s /cm^2^ (G, H). (N = 4-13 mice). (I) Ethology schematic of the black and white box experiment. (J) The proportion of time spent in the white box as a percentage of total time. (N = 3-13 mice). (K) Contents indicated by the dotted boxes in the panel J. (N = 9-13 mice). Data between the four groups at P40. Data are presented as the mean ± SD. *P < 0.05; **P < 0.01; ns, No significance (one-way ANOVA for E-H, J, K).

During the early stage of rd10 mice degeneration, before the substantial loss of photoreceptors, pronounced inflammatory responses coincided with a deterioration in ERG responsiveness 29, 30. The amplitudes of a- and b- waves in rd10 mice declined dramatically compared to those in c57 control mice at P40 (Figure 3A-D). In the plx group, 19 days of continuous microglia depletion (plx40 mice) resulted in significant increases in a- and b-waves at higher light intensities (3.0 cd·s /m^2^) compared to age-matched rd10 mice without a statistical difference at lower light intensities (1.0 cds/m^2^) (Figure 3E-H). In the inter group (replc), the amplitudes of a- and b-waves were significantly higher than those of rd10 (P40) mice (Figure 3B, D, E-H). Specifically, at lower flash intensities (1.0 and 3.0 cd·s /m^2^), rep1c mice showed significantly healthier rod photoreceptors than the other two groups. Nevertheless, at the highest flash intensity (10.0 cd·s /m^2^) that stimulates both the rod and cone, differences in the response were not statistically different (data not shown). These results suggest that Rep-MiG can perform endogenous maintenance of ERG responses, primarily serving to protect rod photoreceptors. Taken together, the first round of Rep-MiG produces a neuroprotective effect and improves the visual function of rd10 mice at specific time points during RD.

In the behavioral test, the white box occupancy time ratio of c57 mice was stabilized around 30% after P21 (Figure 3I, J), yet was markedly increased in the rd10, plx, and inter groups, indicating the compromised discriminating ability between light and dark. In the plx group, only at time points P28 and P33 was the white box occupancy time ratio significantly lower than their contemporaneous rd10 mice. However, the white box occupancy time ratio in the inter group was remarkably reduced compared to that in the rd10 group from P33 to P70 (Figure 3J, K), indicating the preserved visual function in the inter group. These results suggest that Rep-MiG can improve the visual function of rd10 mice.

### Morphological and distribution characteristics of heterogeneous microglia in the retina

**Figure 4:**
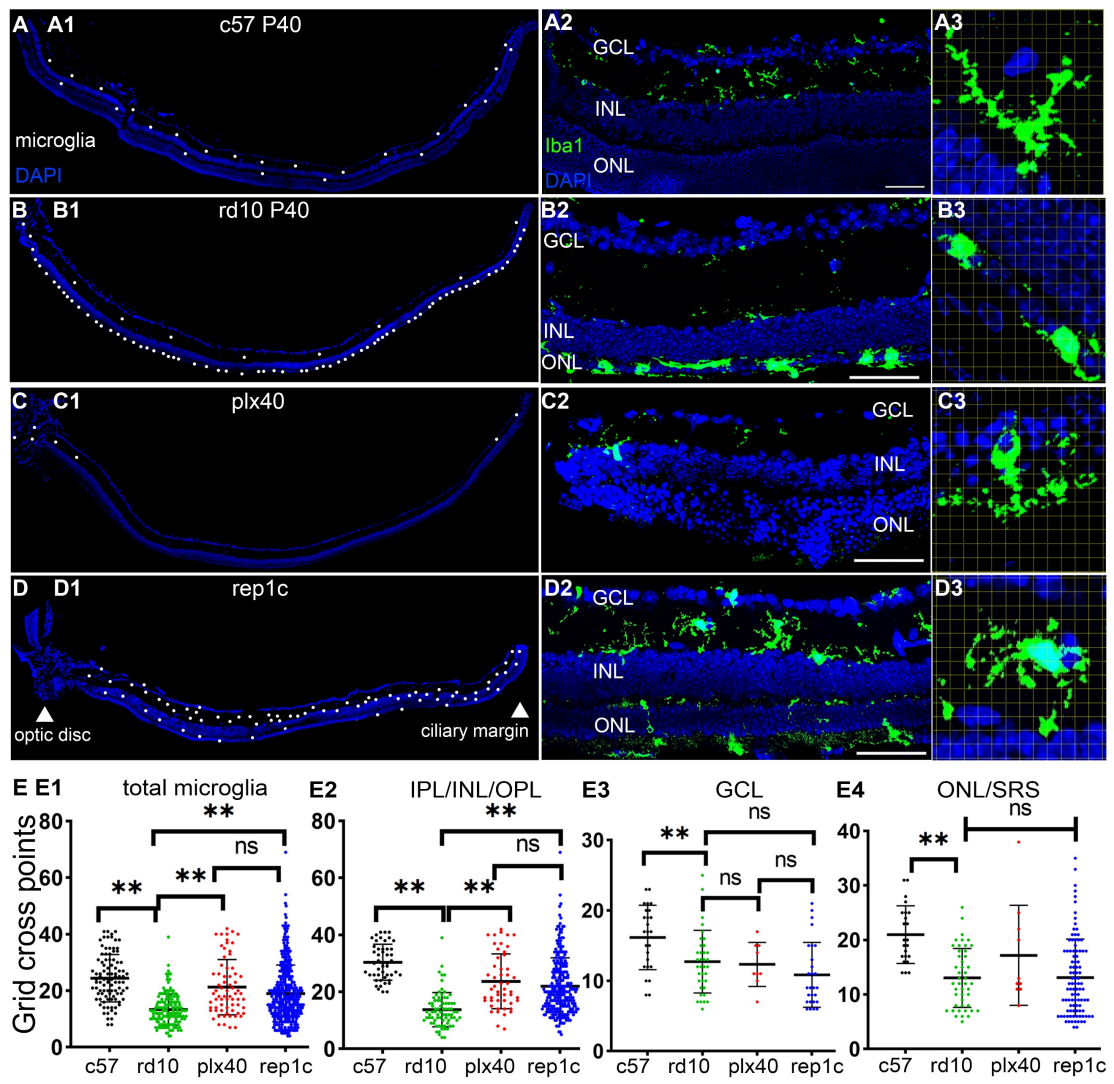
** Morphological and distribution characteristics of heterogeneous microglia in the retinas.** (A-D) Iba1 staining in three groups at different magnifications at P40. (A1-D1) Half retina views with the CMZ on the right and the optic papilla on the left. Each white dot represents a single microglia. (A2-D2) Morphology and distribution of microglia in regional retinal sections. (A3-D3) Grid cross points analysis of microglia (grid length: 14 μm). Representative images of ameboid-like (B3) and ramified microglia (A3, C3, D3). (E1-E4) Statistical analysis of the grid cross points of each microglia in the total retina (E1), inner retina (E3), mid retina (E2), and outer retina (E4) of the c57, rd10, plx, and inter groups at P40. (N = 4 mice, n = 2-3 retinal sections per mouse). Data are presented as the mean ± SD. *P < 0.05; **P < 0.01; ns, No significance (one-way ANOVA for E). Scale bars: 20 μm. GCL: Ganglion cell layer, INL: Inner nuclear layer, ONL: Outer nuclear layer, IPL: Inner plexiform layer, OPL: Outer plexiform layer, SRS: Subretinal space.

To explore the possible mechanism of photoreceptor protection by Rep-MiG, the microglia morphological and distribution characteristics in the three groups were analyzed at P40. In c57 mice at P40, the average number of microglia per retinal section was 18.1, and most were ramified and evenly distributed in IPL and OPL (Figure 4A1-A3). In contrast, in rd10 mice at P40, most microglia had migrated from the inner retina into the ONL and SRS, with a distinctly overactivated amebic-like appearance (Figure 4B1-B3). In plx40 mice, a small number of Dr-MiG started to reappear and were mainly in the IPL around the optic papilla and CMZ. Though ramified, these Dr-MiG tended to be the smaller size with fewer branches (Figure 4C1-C3). In rep1c mice of the inter group, 31.1 ± 7.5 Rep-MiG were counted per retina section and characterized by a ramified appearance and large cell bodies, densely populated in the IPL and OPL throughout the retina (Figure 4D1-D3).

Grid analysis showed that microglia in c57 (P40) mice had more grid cross points than those in rd10 (P40) mice, representing a typical homeostatic phenotype. Dr-MiG in plx40 mice and Rep-MiG in rep1c mice showed more grid cross points than activated microglia in rd10 mice, indicating their ramified morphology (Figure 4E1). This trend was particularly significant in the intermediate retina (IPL/INL/OPL) (Figure 4E2). There were no significant differences (P > 0.05) in grid cross points between the three groups in GCL (Figure 4E3) or the outer retina (ONL and SRS) (Figure 4E4). These results suggest that Dr-MiG and Rep-MiG are more similar to homeostatic microglia in terms of morphology and distribution, implying their ability to reverse the hyperinflammatory microenvironment, activate progenitors, and protect residual photoreceptors.

### Rep-MiG triggered the reprogramming of MG in the retinas of rd10 mice

**Figure 5:**
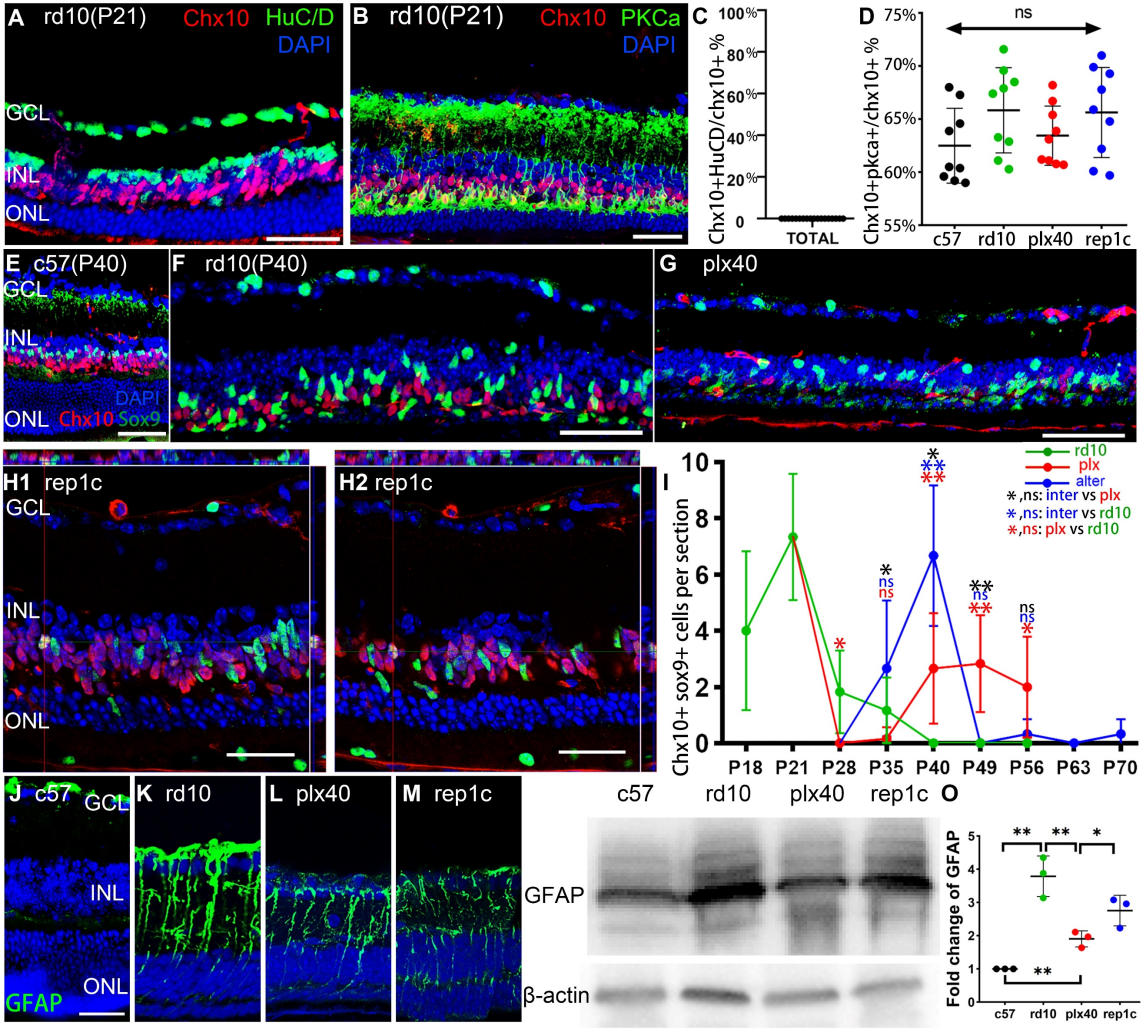
** Rep-MiG triggered MG reprogramming and reduced gliosis of MG.** (A-D) Representative images and statistical analysis of Chx10 staining combined with HuC/D or PKCa staining in retinal sections of the three groups of mice at P40. Graphs C and D show the percentage of Chx10^+^ nuclei to Chx10^+^ HuC/D^+^ nuclei and Chx10^+^PKCa^+^ nuclei, respectively. (N = 3 mice, n = 3 retinal sections per mouse). (E-I) Representative images and statistical analysis of Chx10 staining combined with Sox9 staining in retinal sections of the three groups of mice and c57 mice at P40. Panel I shows the counts of Chx10 colocalized with Sox9 staining in each panoramic retinal section of the three groups at P40. (N = 3 mice, n = 2 retinal sections per mouse). (J-O) GFAP expression was markedly suppressed in the retinas of plx40 and rep1c mice compared to that in rd10 mice at P40. (N = 3 mice). Among them, the retinas of plx40 mice had the lowest GFAP expression. Data are presented as the mean ± SD. *P < 0.05; **P < 0.01; ns, No significance (one-way ANOVA for D, I, O). Scale bars: 40 μm (A, B, E-H, J). GCL: Ganglion cell layer, INL: Inner nuclear layer, ONL: Outer nuclear layer.

To define the Chx10^+^ cells in the retina, we selected several cell markers for double staining, including the amacrine and ganglion cell marker HuC/D and the bipolar cell marker PKCα. No Chx10^+^/HuC/D^+^ cells were observed in any of the groups (Figure 5A, C), whereas nearly 70% of Chx10^+^ could be cytoplasmically enveloped by PKCα, indicating that most Chx10^+^ cells were bipolar (Figure 5B, D). Besides, we confirmed the co-localization of Chx10 and Sox9. In c57 mice, the presence of Sox9^+^/Chx10^+^ cells could not be detected (Figure 5E), implying that in the absence of degeneration, no MG would express markers of neural progenitors. In the rd10 group, at the early stage of retina degeneration (P21), an average of 7.3 Sox9^+^/Chx10^+^ cells were derived from reprogrammed MG in each retinal section image in rd10 mice (Figure 5I). After the rapid degeneration phase (P20-P25), the average number of Sox9^+^/Chx10^+^ cells per retina section sharply declined to less than 2.0 (P28 and P35). No Sox9^+^/Chx10^+^ MG were observed after P40 (Figure 5F, I). In the plx group, after a 7-day microglia elimination (plx28 mice), vast microglia died out, and Sox9^+^/Chx10^+^ cells dropped to 0. However, as Dr-MiG recurred in the plx group at around P40, Sox9^+^/Chx10^+^ MG reappeared nearby (Figure 5G, I). In the inter group, accompanying microglia repopulation, Sox9^+^/Chx10^+^ MG increased significantly compared to contemporaneous rd10 (P40) and plx40 mice. It suggests that Rep-MiG might induce MG reprogramming more markedly than resident microglia and Dr-MiG during degeneration (Figure 5H, I).

During retinal degeneration, MG underwent reactive gliosis, allowing the establishment of glial scars, representing physical and biochemical barriers to regeneration. In this study, GFAP was significantly decreased in the retinas of plx40 and rep1c mice compared to those of rd40 (P40) mice, as shown by immunohistochemistry or quantitative analysis of proteins by western blot (Figure 5J-O). Therefore, Rep-MiG seem to suppress the gliosis of MG and trigger MG reprogramming into retinal progenitors.

**Figure 6:**
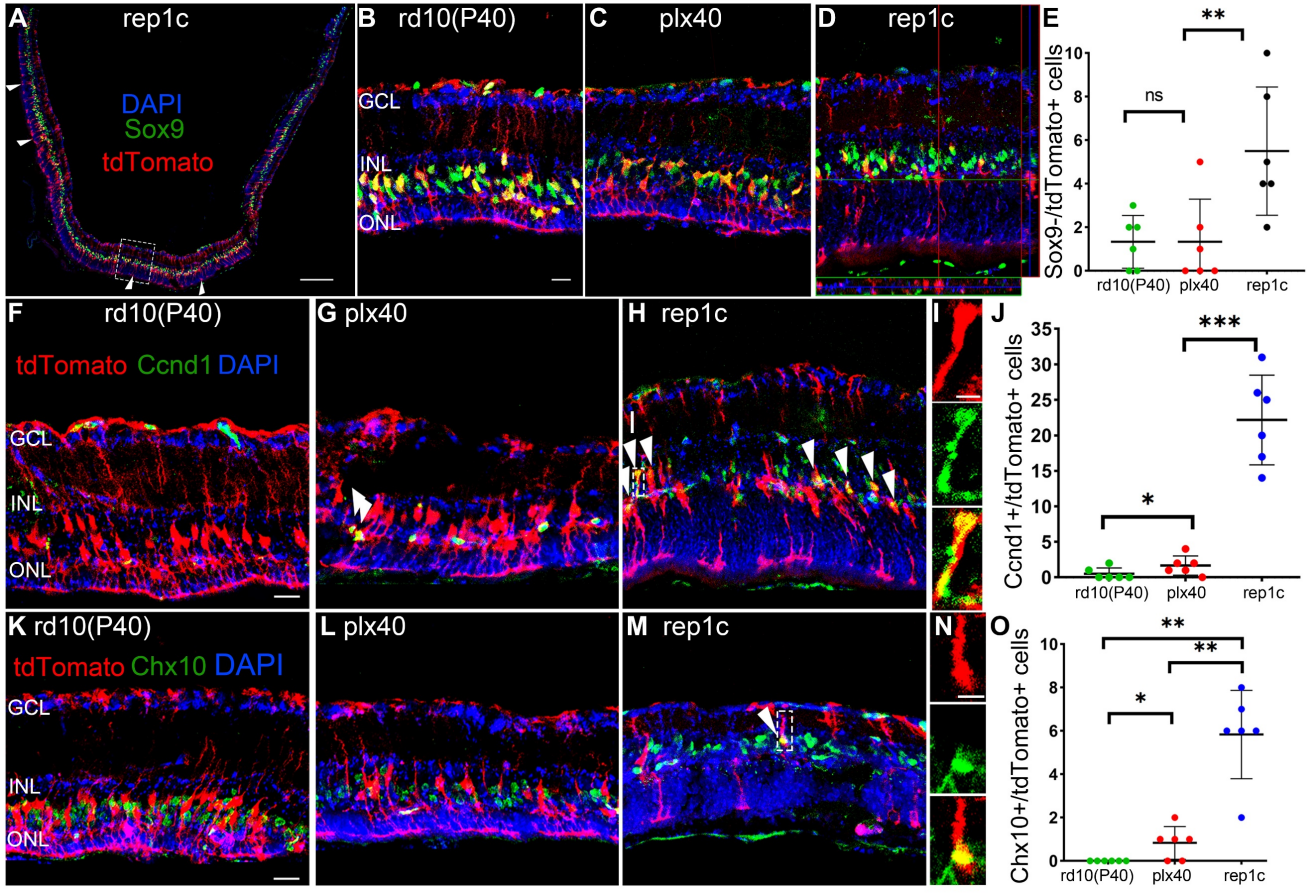
** MG of rep1c mice lost glia identity, proliferated, and expressed retinal progenitor cell markers.** (A) A typical tiled up panoramic view of rep1c mice retinal section with red staining as tdTomato and green staining as Sox9. Each white open arrowhead represented an MG devoid of Sox9^+^ nuclei. See panel D for enlarged images of the dashline box in panel A. (B, C) Typical retinal images for tdTomato and Sox9 of rd10(P40) (B) and plx40 (C) mice. (D) A representative retinal image of rep1c mice with z-axis scanning. (E) The counts of MG nuclei not colocalized with Sox9 in each panoramic retinal section of the three groups at P40. (N = 3 mice, n = 2 retinal sections per mouse). (F-J) Typical retinal images of the three groups of mice at P40, with red staining as tdTomato and green staining as Ccnd1. Each white arrowhead represents an MG with a Ccnd1^+^ nucleus. See panel I for enlarged images of the dashline box in panel H. Panel J shows the counts of MG nuclei colocalized with Sox9 in each panoramic retinal section at P40. (N = 3 mice, n = 2 retinal sections per mouse). (K-O) Typical retinal images of the three groups of mice at P40, with red staining as tdTomato and green staining as Chx10. The white arrowhead represents an MG with a Chx10^+^ nucleus. See panel N for enlarged images of the dashline box in panel M. Panel O shows the counts of MG nuclei colocalized with a Chx10 in each panoramic retinal section at P40. (N = 3 mice, n = 2 retinal sections per mouse). Data are presented as the mean ± SD. *P < 0.05; **P < 0.01; ns, No significance (one-way ANOVA for E, J, O). Scale bars: 200 μm (A), 20 μm (B, F, K), 2 μm (I, J). GCL: Ganglion cell layer, INL: Inner nuclear layer, ONL: Outer nuclear layer.

To confirm our conclusion, cre-loxP^+^ rd10 mice, in which MG expressed tdTomato fluorescent protein specifically, were similarly subjected to the three sets of manipulations described in Figure 1A. We found that the number of Iba1^+^ microglia and Chx10^+^ cells, the ONL thickness, and the changes in electrophysiology or behavioral analysis remained consistent with their corresponding counterparts of rd10 mice. Therefore, we focused on these markers at P40 and found that Sox9^-^/tdTomato^+^ cells significantly increased in the retinas of rep1c mice than those in other two groups of the same age (Figure 6A-E), suggesting that more MG were losing glial markers in the degenerating retina where Rep-MiG appeared. Proliferative MG defined with Ccnd1^+^ and tdTomato^+^, we found that more MG was recruited to the proliferative state in the retinas of rep1c mice (Figure 6F-J). From the overall numbers, there were more tdTomato^+^ cells per retinal section in the rep1c mice than in the mice of the other two groups (data not given). TdTomato^+^ MG expressing Chx10 were increased in the retinas of rep1c mice (Figure 6K-O). Taken together, cre-loxp^+^ rd10 mice, capable of tracing the fate of MG, provided the evidence that an average number of 5.8 MG in each retinal section changed their cell fate from gliosis to reprogramming and entered a progenitor-like proliferative state in the degenerating retina, though most MG failed to reprogram.

### Rep-MiG may contribute to enhancing MG reprogramming by regulating ECM remodeling

To gain the molecular mechanisms underlying the retinal protection and MG -reprogramming conferred by Rep-MiG, RNA-seq analysis was conducted at P40 among the c57, rd10, and rep1c groups. t-SNE plot and heatmap were based on all gene expressions in c57, rd10 and rep1c groups (Figure S3A, B, Table S1). GO enrichment revealed that items related to the photoreceptor and eye development were downregulated in rd10 group (Figure S3C), reflecting the pathophysiological characteristics of the rd10 mice.

**Figure 7:**
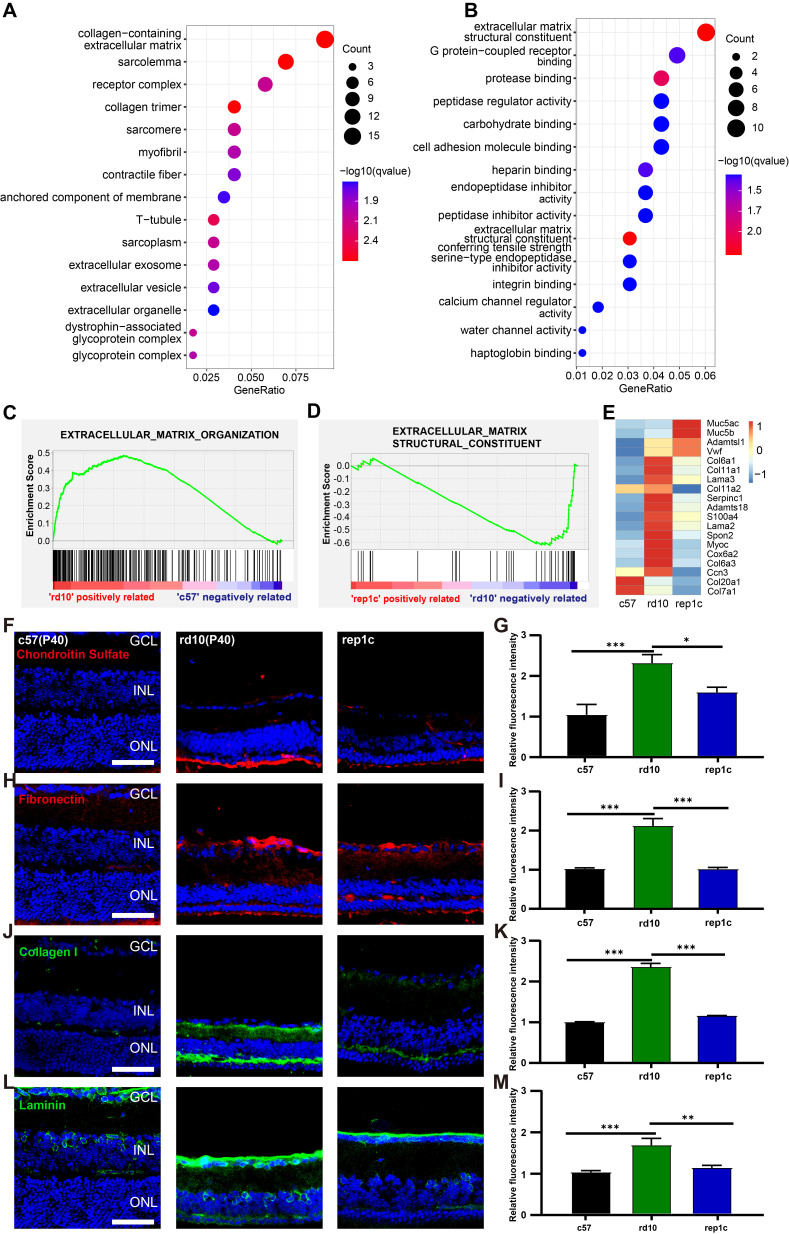
** RNA-seq and IF analysis revealed that Rep-MiG improved ECM remodeling in the degenerative retina.** (A) The 15 most significantly enriched cellular component items in the GO analysis of differentially expressed genes (DEGs) between the rd10 and rep1c groups. (B) The 15 most significantly enriched molecular function items in GO analysis of DEGs between the rd10 and rep1c groups. (C) GSEA for the rd10 and c57 groups showed enrichment of the extracellular matrix organization item. (D) GSEA for the rep1c and rd10 groups showed enrichment of the extracellular matrix structural constituent item. (E) Expression heatmap of extracellular matrix components in the c57, rd10, and rep1c groups. (F) Representative images of chondroitin sulfate staining in the c57, rd10, and rep1c groups. (G) Relative fluorescence intensity analysis of chondroitin sulfate staining. (N = 3 mice, n = 2 images per mouse). (H) Representative images of fibronectin staining in the c57, rd10, and rep1c groups. (I) Relative fluorescence intensity analysis of fibronectin staining. (N = 3 mice, n = 2 images per mouse). (J) Representative images of collagen staining in the c57, rd10, and rep1c groups. (K) Relative fluorescence intensity analysis of collagen staining. (N = 3 mice, n = 2 images per mouse). (L) Representative images of laminin staining in c57, rd10, and rep1c groups. (M) Relative fluorescence intensity analysis of laminin staining. (N = 3 mice, n = 2 images per mouse). Data are presented as the mean ± SD. *P < 0.05; **P < 0.01; ***P < 0.001; Scale bars: 50 μm (F, H, J, L), GCL: Ganglion cell layer, INL: Inner nuclear layer, ONL: Outer nuclear layer.

Next, we performed the comparison between rd10 and inter groups. There were 187 DEGs identified between the rd10 and rep1c groups, of which 67 DEGs were upregulated and 120 DEGs were downregulated in the rep1c group (Supplemental Table 2). Interestingly, GO enrichment analysis of DEGs suggested that ECM-associated pathways were significantly altered following microglia repopulation (Figure 7A, B). Furthermore, GSEA indicated that ECM components were increased in the rd10 group compared to the c57 group (Figure 7C) but reduced in the rep1c group compared to the rd10 group (Figure 7D). The heatmap of ECM-associated DEGs also demonstrated that microglia repopulation reconverted ECM remodeling in the degenerative retina (Figure 7E). Next, immunofluorescence staining of ECM components was used to reveal the alteration in ECM remodeling. Consistent with RNA-Seq screening, immunofluorescence staining showed that ECM components such as chondroitin sulfate (Figure 7F, G), fibronectin (Figure 7H, I), collagen I (Figure 7J, K) and laminin (Figure 7L, M) increased significantly during retinal degeneration compared to the c57 group. Chondroitin sulfate was scarce in the retinas of c57 mice while deposited in the outer layer of ONL in a band in the retinas of rd10 mice. The chondroitin sulfate band of rd10 mice at P40 was thicker and more continuous than c57 mice. However, in rep1c mice, the chondroitin sulfate band was thinner and shorter, showing intermittent distribution, reflected in the intensity analysis (Figure 7F, G). Fibronectin was mainly observed in the GCL, IPL, OPL and ONL of rd10 mice. The expression of fibronectin in rep1c mice was lower than that in rd10 mice (Figure 7H, I). Collagen I was lowly expressed in c57 mice but increased remarkably in rd10 mice, and its dense deposition was in OPL (Figure 7J). The intensity analysis also showed that the expression of collagen I in rep1c mice was reduced compared to that in rd10 mice (Figure 7J, K). Laminin was expressed both in c57 mice and rd10 mice, and was deposited in strips in the GCL, enveloping the nuclei of GCL and INL. Laminin was also deposited in the IPL of rd10 mice, and the expression of rep1c mice was decreased compared to that of rd10 mice (Figure 7L, M). Moreover, GO analysis showed that protease inhibitor activity was significantly enriched (Figure 7B). A serine protease inhibitor, Serpina3n which could improve ECM remodeling and neuro-regeneration 31 was markedly upregulated in the rep1c group (Figure S3H, Table S2). Protease inhibitor activity could promote the re-establishment of microglial homeostasis in a CNS injury model 31. Given that Rep-MiG possess a homeostatic state (Figure 4), it is suspected that the homeostasis of microglia is partly re-established by protease inhibitor activity and that Rep-MiG suppressed ECM remodeling.

**Figure 8:**
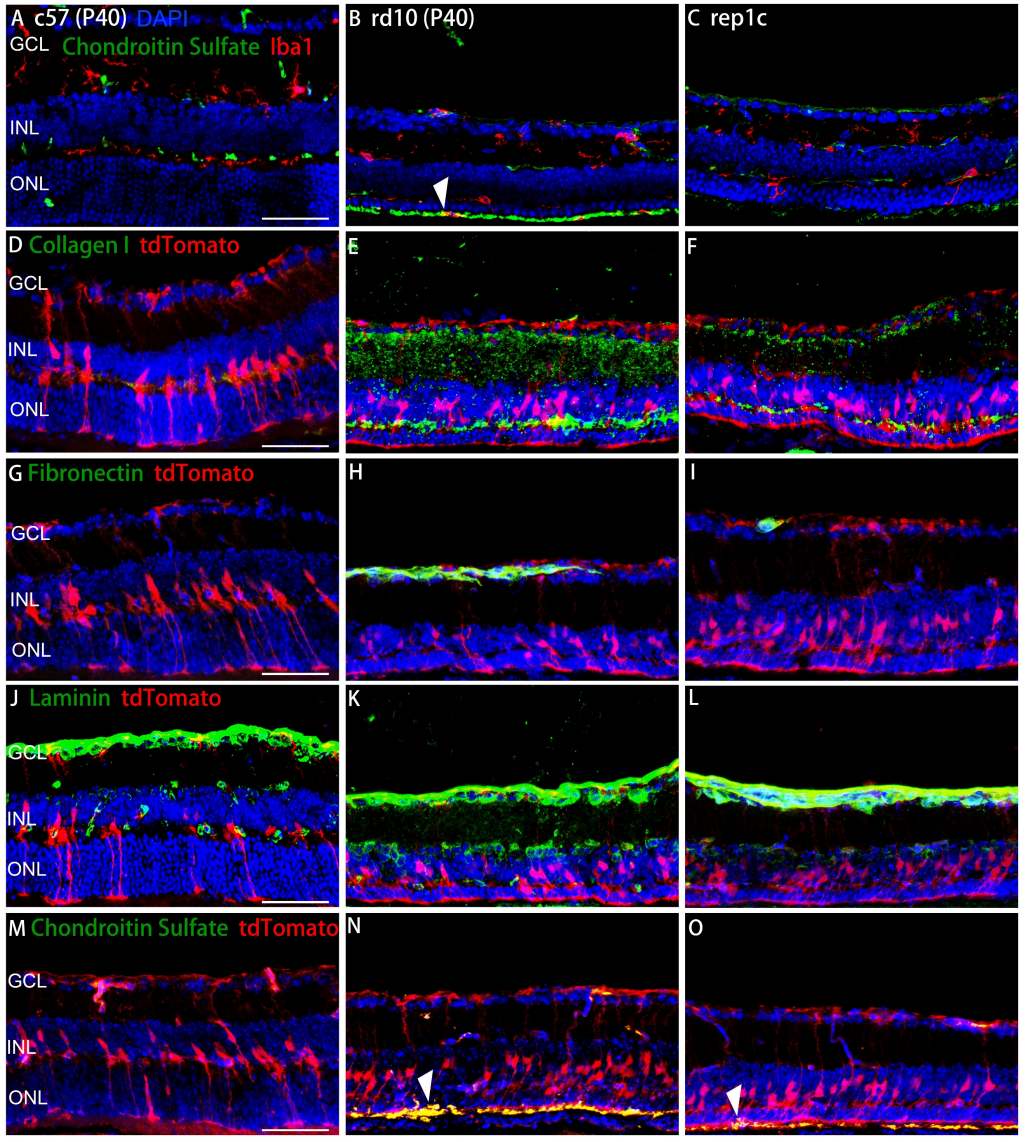
** The spatial relationship between ECM components and microglia or MG.** (A-C) Representative retinal views of mice from the c57, rd10, and rep1c groups with red staining as Iba1 and green staining as chondroitin sulfate. The white arrowhead in panel B shows the colocalization of an Iba1^+^ microglia with chondroitin sulfate located outside the ONL. (D-O) Representative retinal views of mice from the c57, rd10, and rep1c groups showing the spatial relationship between ECM components and MG, with red staining as tdTomato and green staining as collagen I (D-F), fibronectin (G-I), laminin (J-K) and chondroitin sulfate (M-O), respectively. The white arrowheads in panel N and O indicate the colocalization of the end foot of MG with chondroitin sulfate located outside the ONL. Scale bar, 100 μm (A-O). GCL: Ganglion cell layer, INL: Inner nuclear layer, ONL: Outer nuclear layer.

To further investigate the relationship between microglia/MG and ECM, double staining of microglial/MG markers and ECM components were performed. The chondroitin sulfate band was in the outer layer of the ONL and the amoeba-like Iba1^+^ microglia were obvious colocalized with chondroitin sulfate band in rd10 mice (Figure 8B). In rep1c mice, almost no Iba1^+^ microglia were found in the ONL or SRS, perhaps because the outer layer of the microglia was not over-activated, which was consistent with the reduced chondroitin sulfate expression. (Figure 8A-C).

Collagen I enveloped the processes of MG, which implied that the possible role in regulating MG reprogramming (Figure 8D-F). In addition, the colocalization of fibronectin and tdTomato was observed mainly in GCL and OPL in all three groups (Figure 8G-I). As with other ECM components, laminin was observed adjacent to MG and in contact with the MG nuclei (Figure 8J-L). The spatial relationship between chondroitin sulfate and MG was shown in Figure 8M-O. The robust chondroitin sulfate band was colocalized with MG in rd10 mice, while low expression of chondroitin sulfate was observed in rep1c mice (Figure 8M-O). Overall, Rep-MiG regulate the ECM components encircling MG processes, implying that Rep-MiG might attenuate ECM over-deposition, thereby increasing the ratio of MG reprogramming.

GO enrichment analysis showed that eye and visual system development were enriched markedly between the rd10 group and the rep1c group (Figure S3F). Furthermore, GSEA showed upregulated embryonic camera-type eye development in the rep1c group (Figure S3G). The expression of stem cell maintenance- and development-related genes (Figure S3H) was higher in the rep1c group. Additionally, microglia repopulation enabled the decline of inflammatory genes (cd42a, Fcrls, S100a4) and pathways, as well as an increase in anti-inflammatory genes (Lcn2, Pdcd1, Cxcl17) in the rep1c group (Figure S3D, E).

During neurodegenerative diseases, excessive ECM component deposition causes neuroinflammation and glial scarring and prevents neuroregeneration 32, 33. In line with this, regulating the ECM components contributes to solving neuroinflammation and facilitating neuro-regeneration by creating a permissive microenvironment 34. Therefore, we conclude that Rep-MiG aid MG reprogramming partly by adjusting the balance of ECM remodeling, which recapitulates an embryonic developmental state in the degenerative retina.

## Discussion

Recently, the role of microglia in neurogenesis has been revealed in the degenerative brain and retina. However, the effects of different microglia subsets on stem cells remain unknown. In this study, we demonstrated that microglia with different phenotypes presented various roles in stem cell-like property activation. Rep-MiG increased the number of retinal progenitors and promoted MG reprogramming compared to activated microglia during photoreceptor degeneration. The effect on MG reprogramming may explain why Rep-MiG preserve photoreceptor degeneration and visual function. Furthermore, we suspect these Rep-MiG may suppress ECM remodeling to contribute to MG reprogramming. Consequently, our results provide a new therapeutic strategy for retinal degeneration.

Microglia are recognized as the critical regulators during RP, and CSF1R inhibitors offer an excellent tool for revealing the roles of microglia and modulating microglia behavior. Here, plx3397, a selective CSF1R kinase inhibitor, was applied to clear retinal microglia. After plx3397 withdrawal, repopulating microglia replenished the entire retina rapidly from two origins. The majority originated from the optic nerve in the central retina, and a few were derived from the macrophages in the ciliary body/iris in the peripheral retina, which coincides with the findings of previous studies 20, 35. In the degenerative microenvironment, some Rep-MiG progressively assimilated into an activated phenotype migrated toward the outer retina, and acquired an amebic-like profile. However, most Rep-MiG remained in the inner retina with ramified morphology at P40. These findings imply that Rep-MiG may be homeostatic, anti-inflammatory, and neuroprotective phenotypes during the first round of MiG recolonization in the degenerative retina.

Remarkably, for the first time, we demonstrated that Rep-MiG instead of microglial elimination improved visual function and visual behavior in the degenerative retina. Microglial repopulation can promote functional recovery by solving neuroinflammation and sculpting the synaptic landscape 22, 23. In Alzheimer's disease models, repopulated microglia could reverse the disease-associated microglia phenotype and recover a resting phenotype 40, consistent with our results. Besides, microglial repopulation modulated the microenvironment of the injured brain, exerted a neuroprotective effect via interleukin-6 signaling, and alleviated cognitive and learning deficits. However, several groups have reported that eliminating activated microglia rather than microglial repopulation could exert a neuroprotective effect during neurodegeneration 36, 37. Unfortunately, the mechanisms responsible for this difference or controversy are unclear and require further study.

Additionally, we found that Chx10^+^ cells increased significantly at the beginning of retinal degeneration in rd10 mice before declining, indicating that the acute response to injury activated the stem cell properties in the retina but failed to repair the retina eventually. In our previous study, we noticed that the microglia phenotype changed according to the development of photoreceptor degeneration in RCS rats 14. In the present study, the number of Chx10^+^ cells also fluctuated with microglial clearance and repopulation in the inter group. Following the withdrawal of plx3397, the spatial distribution of numerous re-emerged Chx10^+^ cells was strongly associated with the Rep-MiG. Interestingly, although substantial ameboid microglia were present in ONL and SRS of the rd10 mice at P49 and P56, the number of Chx10^+^ cells remained low. It suggested that Rep-MiG supported the maintenance of a stem cell-like phenotype. The spatial relationship between Chx10^+^ cells and microglia also implied that the effect of Rep-MiG on activating stem cell properties might result from direct contact or paracrine actions. Similarly, remyelination in the brain requires microglial necrosis and repopulating to the pro-regenerative phenotype 38. Rep-MiG stimulated adult neurogenesis in the hippocampus of the traumatic brain injury model via interleukin-6 trans-signaling 39.

Crosstalk between microglia and MG contributes to neuroinflammation, scar formation, and MG-mediated retinal neuroregeneration 19, 29, 40. In the present study, retinal gliosis reached the highest level in the rd10 mice but less in the plx40 and rep1c group. Interestingly, the GFAP level in plx40 mice retinas was significantly lower than that in rep1c mice, which was contrary to our expectation but was explainable. As shown in Figure 4, only a few ramified Dr-MiG distributed in the IPL were sporadically seen in the retinas of plx40 mice, with no ameboid shape indicative of an over-activated microglia state. Thus, the contribution of microglia in promoting MG gliosis was relatively low. In rep1c mice, 21.3% of all Rep-MiG were in the outer retina, with their amebic shapes indicating their activated status. These cells would make corresponding contributions to gliosis. The number of Chx10^+^/Sox9^+^ cells derived from the reprogrammed MG in the inter group was significantly increased compared to that in the rd10 and plx groups, suggesting that Rep-MiG promoted MG reprogramming. Previous studies have shown that in the injured retina of zebrafish, MG undergo a process of dedifferentiation, during which MG progressively lose the expression of Sox9. Thus, a more precise and convincing means to determine the fate of MG in degenerating retina is required. The reprogramming of MG was further confirmed using cre-conditional reporter mice in which MG specifically expressed tdTomato fluorescent protein.

The results of RNA-Seq showed significant changes in ECM-associated pathways following microglia repopulation. Following neural injury, the dynamic structural network of the ECM is disrupted, usually leading to excessive deposition of ECM 41, 42. The anomalous deposition of ECM triggered a pro-inflammatory microenvironment coincided with increased extracellular space stiffness, resulting in scar formation and retinal fibrosis 42. These changes form an inhibitory physicochemical barrier, aggravate disease progression, and impede neuroregeneration 32, 43, 44. Here, ECM remodeling was observed, characterized by ECM over-deposition in the degenerative retina. Recently, several groups have reported that microglia have critical implications for ECM remodeling by synthesizing, secreting, engulfing, and degrading ECM components 45, 46. Here, we showed for the first time that Rep-MiG suppressed ECM remodeling. The protease inhibitor activity was essential for microglial homeostasis re-establishment and regulating ECM remodeling in a nerve injury model 31. Notably, protease inhibition activity-associated pathways and Serpina3n, an important protease inhibitor of microglia-mediated ECM regulation, were upregulated in the inter group. Thus, it is speculated that Rep-MiG may reestablish homeostasis via protease inhibition signaling, and suppress ECM remodeling for contributing to neuronal survival and neuroinflammation alleviation. ECM molecules play pivotal roles in neurogenesis and neuroregeneration by regulating neural stem cell fate 43, 47. Regulating ECM remodeling alleviates neuroinflammation, retards neurodegeneration, and promotes neuroregeneration in the CNS 34, 42. Regulating ECM remodeling in the retina could alleviate glial scar and benefit the integration, migration, and differentiation of transplanted stem cells 48. Moreover, a notch pathway inhibitor has been shown to reduce ECM protein expression and MG gliosis 49. Knockout of the ECM molecule Tenascin C affected the proliferation of MG 50. Furthermore, degrading the ECM through Gelatinases could promote MG reprogramming in the chicken retina 51. Here, we found that microglia repopulation suppressed ECM remodeling and reprogrammed MG in the degenerative retina, protecting the photoreceptors and improving visual function. In addition, ECM components could be colocalized with microglia and MG, indicating that Rep-MiG could regulate the ECM remodeling, and the suppressed ECM remodeling might facilitate MG reprogramming.

Interestingly, it has also been revealed that Rep-MiG could maintain homeostatic characteristics, possibly due to the creation of an embryonic development state. Previous studies have reported that in the newborn spinal cord injury mouse model, microglia could return to homeostasis soon through peptidase inhibitors, then mediate scar-free spinal cord repair 31. Another study compared the gene expression profile during development and regeneration in the axolotl brain and found that the regenerated cells had a similar change process with the embryonic development cells 52. A recent study found that maternal immune activation decreased the immune reactivity of the offspring microglia but increased the activity of astrocytes. Microglia repopulation could reverse the above phenomena 53. These findings suggested that microglia affected in early development might change the immune state, while microglia repopulation could restore the immune reactivity to a normal state. It implies that Rep-MiG probably are similar to microglia of embryonic development, which reconstructs the normal immune microenvironment. Our results showed that Rep-MiG maintained homeostatic characteristics in the degenerated retina and promoted gene expressions related to anti-inflammation, neuroprotection, development, and stem cell. Meanwhile, a high expression of progenitor marker Chx10 coincided with the presence of Rep-MiG. Therefore, we speculate that Rep-MiG recapitulate an embryonic developmental state in the degenerative retina, which is of great significance for the protection of retinal neurons.

In summary, Rep-MiG showed homeostatic characteristics and suppressed ECM remodeling, which might promote MG reprogramming and stimulate the stem cell property. Consequently, Rep-MiG could protect retinal photoreceptors and improve visual function during retinal degeneration. Thus, our study provides a potential strategy for treating retinitis pigmentosa.

## Supplementary Material

Supplementary figures.

## Figures and Tables

**Table 1:** Antibodies used for immunofluorescence (IF) staining

Name	Application	Host	Supplier
Primary** antibodies**			
PKCa	IF(1:500)	Rabbit	Abcam
Ibal	IF(1:500)	Rabbit	Wako
Sox9	IF(1:500)	Rabbit	Abcam
GAPDH	WB(1:2000)	Mouse	Sigma
PAX6	IF(1:500)	Mouse	Abcam
Chx10	IF(1:300)	Mouse	Santa Cruz
GFAP	IF(1:500)/WB(1:2000)	Rabbit	Abcam
Fibronectin	IF(1:300)	Mouse	Abcam
Fibronectin	IF(1:300)	Rabbit	Abcam
Chondroitin	IF(1:300)	Mouse	Abcam
Collagen I	IF(1:300)	Rabbit	Abcam
Laminin	IF(1:300)	Rabbit	Abcam
Secondary antibodies			
Anti-mouse IgG Alexa Fluor®568	IF(1:500)	Goat	Thermo Fisher Scientific
Anti-rabbit IgG Alexa Fluor®568	IF(1:500)	Goat	Thermo Fisher Scientific
Anti-mouse-IgG Alexa-Fluor-488	IF(1:500)	Goat	Thermo Fisher Scientific
Anti-rabbit-IgG Alexa-Fluor-488	IF(1:500)	Goat	Thermo Fisher Scientific
Anti-rabbit-IgG Alexa-Fluor-647	IF(1:500)	Goat	Thermo Fisher Scientific
Anti-Goat-IgG Alexa-Fluor-488	IF(1:500)	Donkey	Life technologies
Anti-mouse IgG HRP	WB (1:2000)	Goat	Beyotime
Anti-rabbit IgG HRP	WB (1:2000)	Goat	Beyotime
