# Supplementary material for: Repopulated retinal microglia promote Müller glia reprogramming and preserve visual function in retinal degenerative mice
Source: Theranostics. 2023 Mar 13;13(5):1698–715. doi: 10.7150/thno.79538 (PMC10086209; doi:10.7150/thno.79538)
Supplement: Supplementary file 1 — Supplementary figures. [file thnov13p1698s1.pdf]

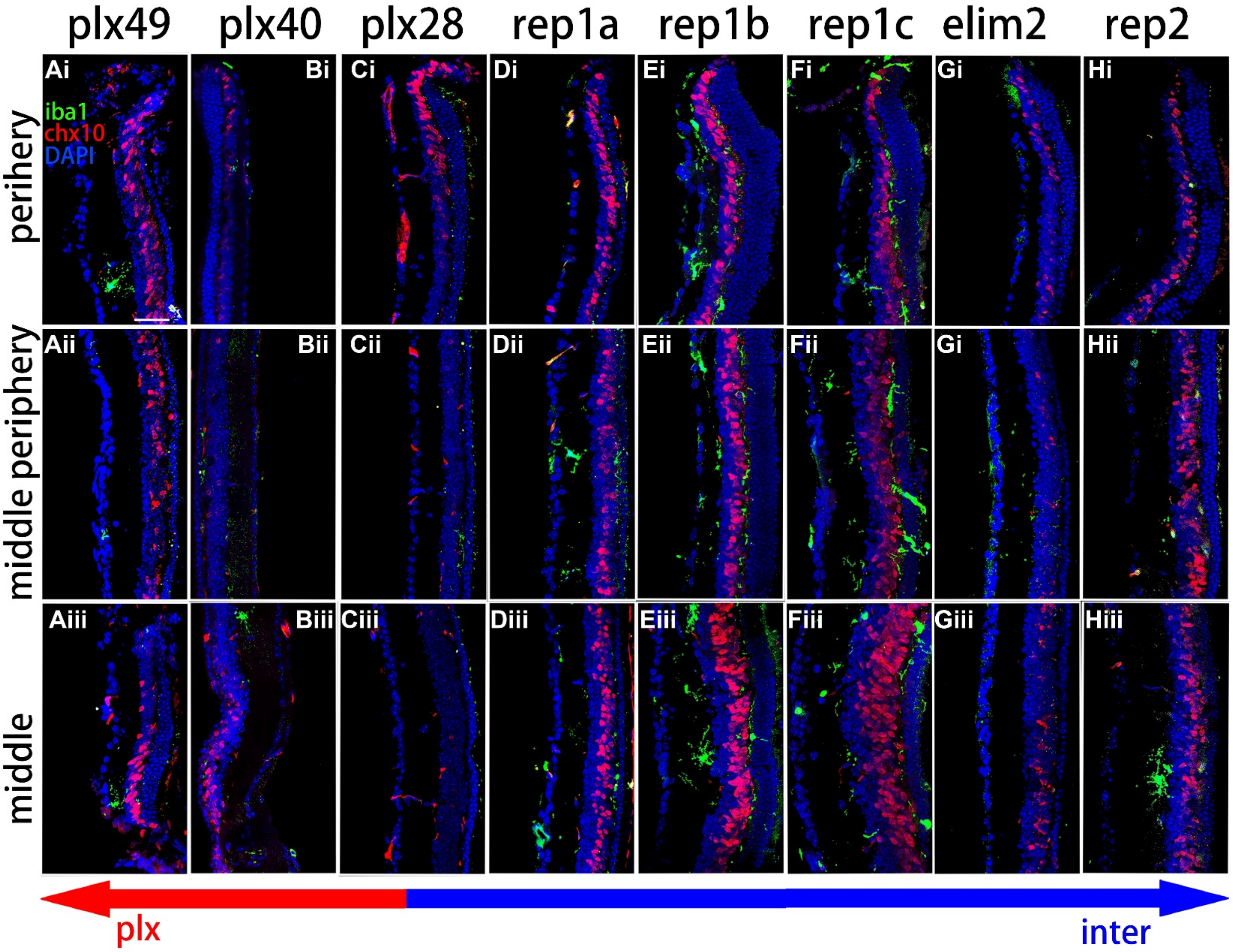

Figure S1. Spatial distribution of Iba1 and Chx10 staining of retinal sections from plx and inter groups

Panel C is a representative mouse retina from the plx28 group of rd10 mice that underwent 7-day plx3397 administration (350 ppm) starting at p21. The two treatments starting from the plx28 group resulted in two outcomes (plx groups to the left and inter groups to the right). The first row of panels (i) is the ciliary margin of a retina from each group, the second row of panels (ii) is the mid-peripheral portion of a retina from each group, and the third row of panels (iii) is the optic papilla or areas close to it for each group. Scale bar: 100 µm.

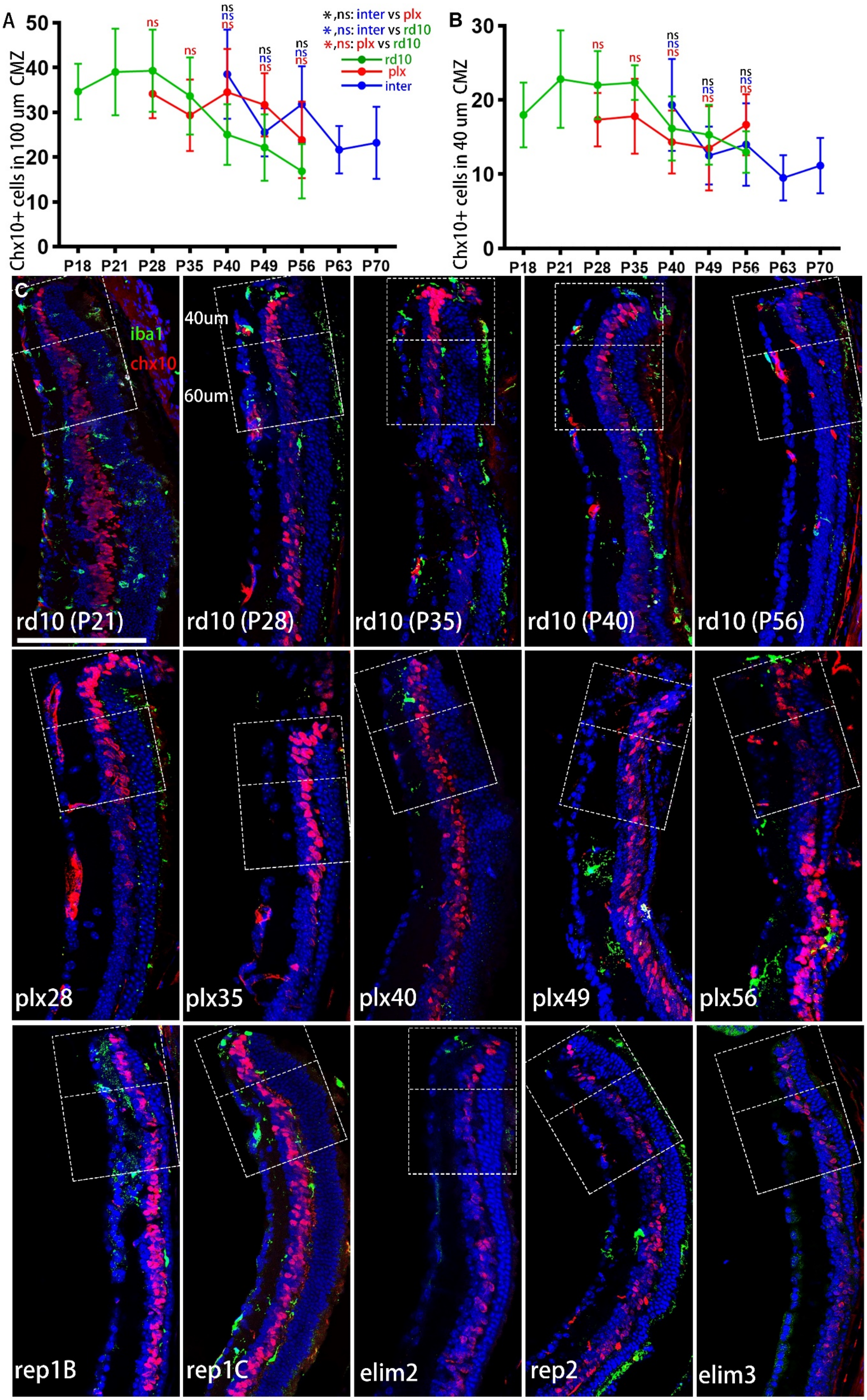
A
B
C
*,ns: inter vs plx
*,ns: inter vs rd10
*,ns: plx vs rd10
rd10
plx
inter
Chx10+ cells in 100 um CMZ
Chx10+ cells in 40 um CMZ
P18
P21
P28
P35
P40
P49
P56
P63
P70
ns
iba1
chx10
40um
60um
rd10 (P21)
rd10 (P28)
rd10 (P35)
rd10 (P40)
rd10 (P56)
plx28
plx35
plx40
plx49
plx56
rep1B
rep1C
elim2
rep2
elim3

Figure S2. Chx10 + cell counts in the retinal CMZ

(A, B) Retinal sections were immune-stained, panoramic photographed, and tiled up for the rd10, plx, and inter groups. Chx10 + cells were counted in the ciliary marginal zone (100 μm or 40 μm ends of each retinal section) of each retina (rd10 green, plx red, inter, blue). (N = 3 mice, n = 2 images per mouse).

(C) Representative retinal sections from each of the three groups, with areas in the white box outlining the ciliary marginal zone.

Data are presented as the mean ± SD. *P < 0.05; **P < 0.01; ns, No significance (one-way ANOVA). Red, blue, and black asterisks or ns represent differences in the significance of the data of the plx group vs the rd10 group, the inter group vs the rd10 group, and the inter group vs the plx group. Scale bar, 200 μm (B).

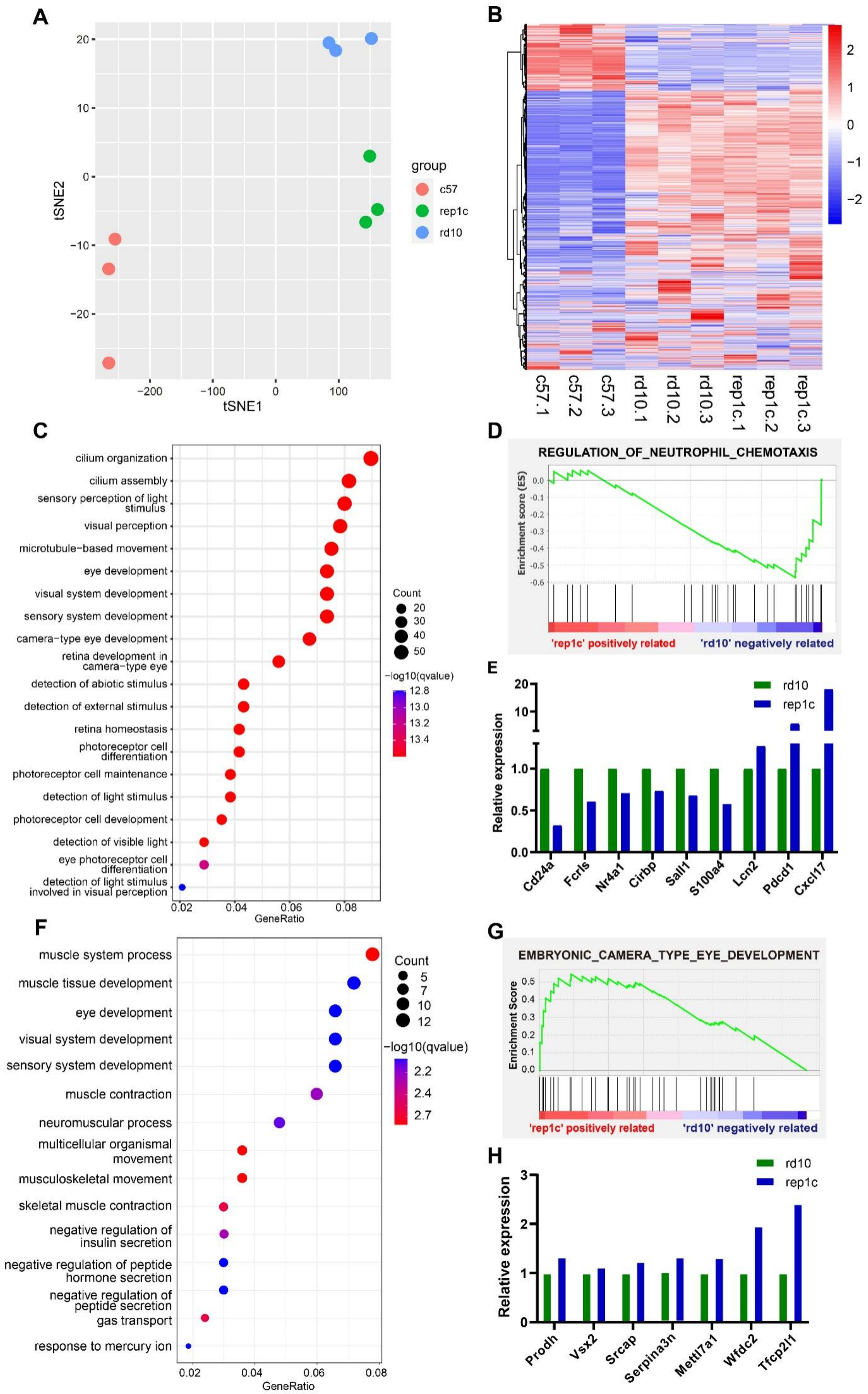

A
tSNE2
tSNE1
group
c57
rep1c
rd10
20
10
0
−10
−20
−200
−100
0
100
B
2
1
0
−1
−2
c57.1
c57.2
c57.3
rd10.1
rd10.2
rd10.3
rep1c.1
rep1c.2
rep1c.3
C
cilium organization
cilium assembly
sensory perception of light stimulus
visual perception
microtubule−based movement
eye development
visual system development
sensory system development
camera−type eye development
retina development in camera−type eye
detection of abiotic stimulus
detection of external stimulus
retina homeostasis
photoreceptor cell differentiation
photoreceptor cell maintenance
detection of light stimulus
photoreceptor cell development
detection of visible light
eye photoreceptor cell differentiation
detection of light stimulus involved in visual perception
GeneRatio
0.02
0.04
0.06
0.08
Count
20
30
40
50
−log10(qvalue)
12.8
13.0
13.2
13.4
D
REGULATION_OF_NEUTROPHIL_CHEMOTAXIS
Enrichment score (ES)
0.0
-0.1
-0.2
-0.3
-0.4
-0.5
-0.6
'rep1c' positively related
'rd10' negatively related
E
Relative expression
rd10
rep1c
20
10
1.0
0.5
0.0
Cd24a
Fcrls
Nr4a1
Cirbp
Sall1
S100a4
Lcn2
Pdcd1
Cxcl17
F
muscle system process
muscle tissue development
eye development
visual system development
sensory system development
muscle contraction
neuromuscular process
multicellular organismal movement
musculoskeletal movement
skeletal muscle contraction
negative regulation of insulin secretion
negative regulation of peptide hormone secretion
negative regulation of peptide secretion
gas transport
response to mercury ion
GeneRatio
0.02
0.04
0.06
0.08
Count
5
7
10
12
−log10(qvalue)
2.2
2.4
2.7
G
EMBRYONIC_CAMERA_TYPE_EYE_DEVELOPMENT
Enrichment Score
0.5
0.4
0.3
0.2
0.1
0.0
'rep1c' positively related
'rd10' negatively related
H
Relative expression
rd10
rep1c
3
2
1
0
Prodh
Vsx2
Srcap
Serpina3n
Mettl7a1
Wfdc2
Tfcp2l1

Figure S3. Transcriptomic sequencing in the c57, rd10 and rep1c groups

(A) t-SNE plot showing the overall presentation of data in all three groups.
(B) Expression of heatmap based on all genes in the c57, rd10, and rep1c groups
(C) The 20 most significantly enriched items in the GO analysis of low expression genes in the rd10 group compared to the c57 group.
(D) GSEA between the rep1c and rd10 groups showed enrichment of the regulation of neutrophil chemotaxis item.
(E) FPKM relative expression of DEGs associated with inflammation (i.e., cd42a, Fcrls, and S100a4) and anti-inflammation (Lcn2, Pdcd1, Cxcl17) in the rd10 and rep1c groups.
(F) The 15 most significantly enriched biological process items in the GO analysis of differentially expressed genes (DEGs) between the rd10 and rep1c groups.
(G) GSEA between the rep1c and rd10 groups showed the enrichment of an embryonic camera-type eye development item.
(H) FPKM relative expression of DEGs associated with neuroregeneration and stem cell maintenance in the rd10 and rep1c groups.
